# Interleukin-15 stimulates natural killer cell-mediated killing of both human pancreatic cancer and stellate cells

**DOI:** 10.18632/oncotarget.18185

**Published:** 2017-05-25

**Authors:** Jonas R.M. Van Audenaerde, Jorrit De Waele, Elly Marcq, Jinthe Van Loenhout, Eva Lion, Johan M.J. Van den Bergh, Ralf Jesenofsky, Atsushi Masamune, Geert Roeyen, Patrick Pauwels, Filip Lardon, Marc Peeters, Evelien L.J. Smits

**Affiliations:** ^1^ Center for Oncological Research, Faculty of Medicine and Health Sciences, University of Antwerp, Antwerp, Belgium; ^2^ Laboratory of Experimental Hematology, Faculty of Medicine and Health Sciences, University of Antwerp, Antwerp, Belgium; ^3^ Department of Medicine II, Medical Faculty of Mannheim, University of Heidelberg, Mannheim, Germany; ^4^ Division of Gastroenterology, Tohoku University Graduate School of Medicine, Sendai, Japan; ^5^ Department of Hepatobiliary, Endocrine and Transplantation Surgery, Antwerp University Hospital, Antwerp, Belgium; ^6^ Department of Pathology, Antwerp University Hospital, Antwerp, Belgium; ^7^ Department of Oncology, Multidisciplinary Oncological Centre Antwerp, Antwerp University Hospital, Antwerp, Belgium

**Keywords:** natural killer (NK) cells, pancreatic cancer (PDAC), pancreatic stellate cells (PSC), immunotherapy, interleukin-15 (IL-15)

## Abstract

Pancreatic ductal adenocarcinoma (PDAC) is the 4^th^ leading cause of cancer-related death in Western countries with a 5-year survival rate below 5%. One of the hallmarks of this cancer is the strong desmoplastic reaction within the tumor microenvironment (TME), orchestrated by activated pancreatic stellate cells (PSC). This results in a functional and mechanical shield which causes resistance to conventional therapies. Aiming to overcome this resistance by tackling the stromal shield, we assessed for the first time the capacity of IL-15 stimulated natural killer (NK) cells to kill PSC and pancreatic cancer cells (PCC). The potency of IL-15 to promote NK cell-mediated killing was evaluated phenotypically and functionally. In addition, NK cell and immune checkpoint ligands on PSC were charted. We demonstrate that IL-15 activated NK cells kill both PCC and PSC lines (range 9-35% and 20-50%, respectively) in a contact-dependent manner and significantly higher as compared to resting NK cells. Improved killing of these pancreatic cell lines is, at least partly, dependent on IL-15 induced upregulation of TIM-3 and NKG2D. Furthermore, we confirm significant killing of primary PSC by IL-15 activated NK cells in an *ex vivo* autologous system. Screening for potential targets for immunotherapeutic strategies, we demonstrate surface expression of both inhibitory (PD-L1, PD-L2) and activating (MICA/B, ULBPs and Galectin-9) ligands on primary PSC. These data underscore the therapeutic potential of IL-15 to promote NK cell-mediated cytotoxicity as a treatment of pancreatic cancer and provide promising future targets to tackle remaining PSC.

## INTRODUCTION

Pancreatic Ductal Adenocarcinoma (PDAC) is a devastating disease with a 5-year survival rate below 5%, rendering it the 4^th^ most common cause of cancer-related death worldwide [1–3]. For the past 20 years, PDAC patients who are not eligible for surgical resection – covering 85% of the population – receive first line treatment with gemcitabine [3–5]. Despite this treatment, survival outcomes remain poor with less than 6 months for metastatic patients and 9-12 months for patients with locally advanced disease [6, 7]. New chemotherapeutic combinations like FOLFIRINOX (leucovorin, 5-fluorouracil, irinotecan and oxaliplatin) have a limited impact on survival, leaving PDAC patients in desperate need of new treatment options [8–10].

A hallmark of PDAC is the strong desmoplastic reaction which occurs in the tumor microenvironment (TME) resulting in a dense fibrotic/desmoplastic stroma that surrounds the pancreatic cancer cells (PCC) [11–13]. This stroma, which can cover more than 50% of the tumor, acts as a mechanical and functional shield causing a diminished delivery of systemically administered anticancer agents due to a high intratumoral pressure and low microvascular density [11–15]. The main orchestrators of this phenomenon are the activated pancreatic stellate cells (PSC). These myofibroblast-like cells enhance the development, progression and invasion of PDAC through their extensive crosstalk with the PCC, resulting in reciprocal stimulation and therapy resistance. It is strongly believed that (new) treatment options will only be able to exert their full potential provided that this shield is breached [11–14, 16, 17].

In several cancer types immunotherapy has prevailed where more conventional therapies have failed. In 2015, the Cancer Immunotherapy Trials Network (CITN) ranked the immune checkpoint inhibitors targeting programmed death (PD-)1 or its ligand PD-L1 as the number 1 on the priority list for immunotherapeutic agents [18]. These blocking antibodies have taken a leap forward in the struggle against several cancers, including melanoma and lung cancer [19, 20]. Unfortunately, despite the fact that PD-1 and PD-L1 are expressed in PDAC, anti-PD-1 therapy achieved only little effect [7, 21].

Interleukin (IL)-15, ranked third on the CITN priority list [18], is a highly attractive cytokine for immune cell stimulation because it can increase the proliferation and persistence of natural killer (NK) and T cells but does not – in contrast to IL-2 – stimulate immune-suppressing regulatory T cells (Tregs) [22]. The majority of (novel) immunotherapeutic strategies focuses on activating T cells. However, innate immune cells – especially NK cells – are less often suppressed by current chemotherapeutic treatments and are demonstrated attractive effectors in immunotherapeutic strategies [23, 24], since one of their main tasks is to target cancer cells [25]. In PDAC specifically, it has been shown that higher absolute levels of NK cells in circulation are associated with better survival [26]. Along the same lines, NKG2D – one of the major activating receptors of NK cells – is shown to be reduced on NK cells in PDAC patients [27]. Here, we are the first to look into the therapeutic potential of IL-15 stimulated NK cells in PDAC. The aim of this study was to evaluate the potency and mechanism of IL-15 to promote NK cell-mediated killing of PCC and PSC, the latter in order to breach the stromal shield surrounding the tumor rendering the tumor more susceptible for anticancer agents. Additionally, we screened PSC for a range of prototypic NK cell and highly attractive immune checkpoint ligands with a view to grow our knowledge on novel therapeutic targets.

## RESULTS

### IL-15 potentiates NK cells from healthy donors to kill PCC and PSC lines

**Figure 1:**
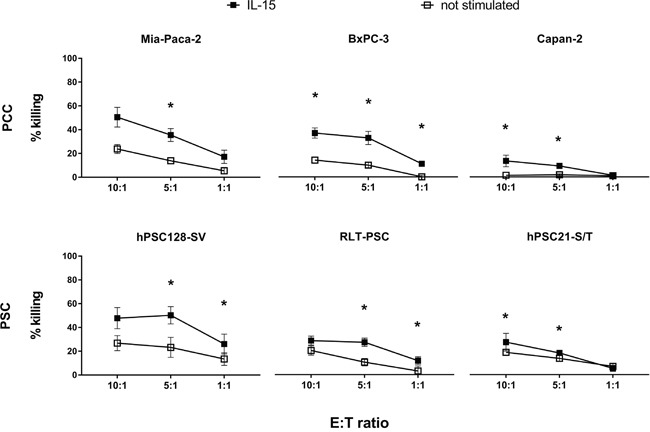
NK cell-mediated killing of PCC and PSC lines Percentages of killing of three PCC lines (upper graphs) and three PSC lines (lower graphs) by purified healthy unstimulated (white) and IL-15 stimulated (black) NK cells at different E:T ratios: 10:1, 5:1 and 1:1 after a 4-hour flow cytometric cytotoxicity assay are shown. Data is depicted as mean (± SEM) for five independent donors. *, p < 0.05. Wilcoxon Signed Ranks Test (2-tailed).

Depicted in Figure 1, purified unstimulated NK cells from healthy donors are capable of killing 2 out of 3 PCC lines (Mia-Paca-2 and BxPC-3; no killing of Capan-2) and all 3 PSC lines (hPSC128-S/V, RLT-PSC and hPSC21-S/T). Stimulation of NK cells with IL-15 clearly enhances NK cell-mediated cytotoxicity for all PCC and PSC lines tested with a statistically significant increase (range 1.3 to 5.3-fold) for all cocultures in the NK cell:target cell ratio 5:1. The dose-response analysis reveals that the 1:1 ratio has a lower amount of killing, whereas there is no general difference in killing between the ratios 10:1 and 5:1.

### Direct cell-cell contact between NK cell and PCC/PSC is necessary for killing

**Figure 2:**
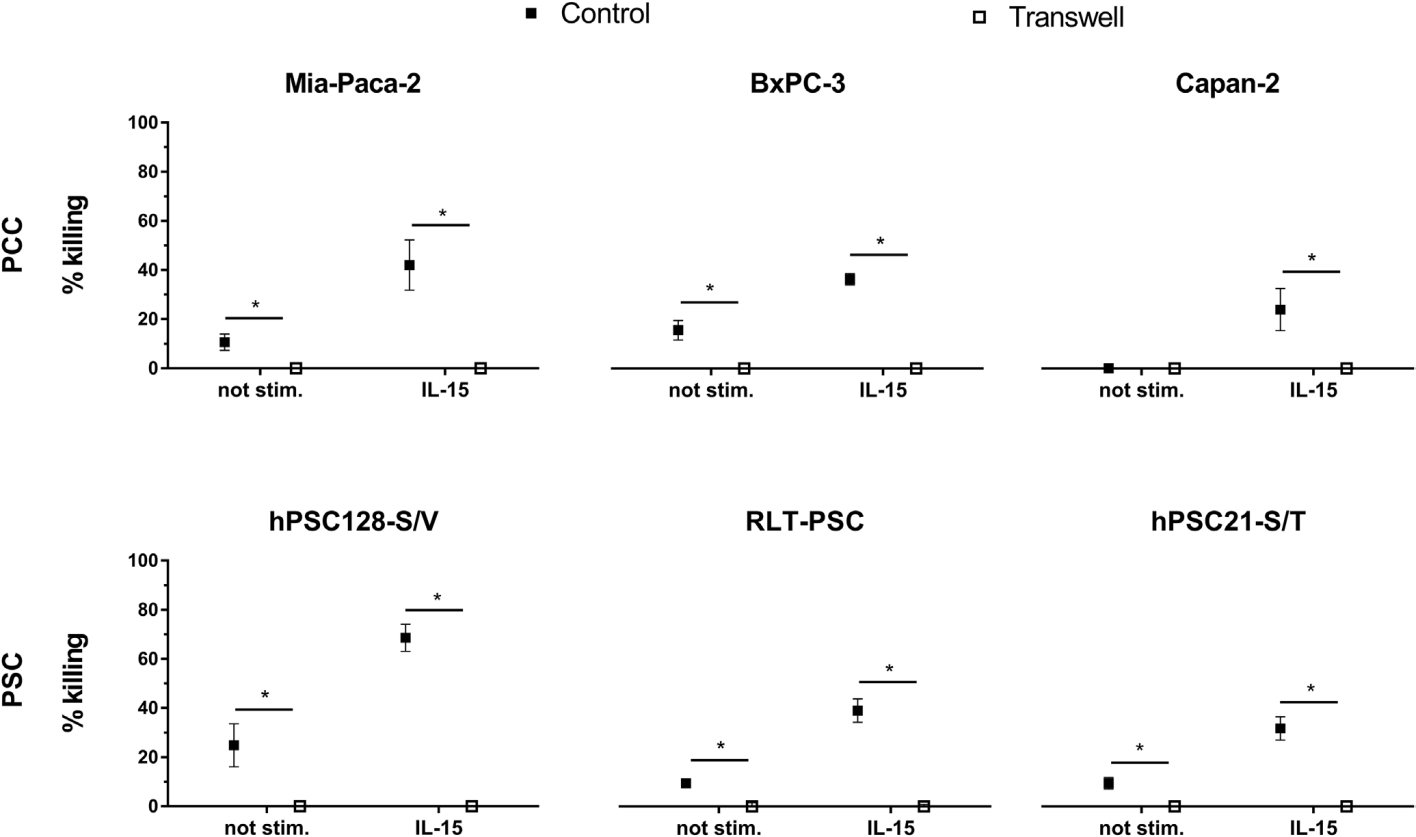
NK cell-mediated killing of PCC and PSC is contact-dependent Killing percentages are shown with (un)stimulated NK cells for the PCC lines Mia-Paca-2, BxPC-3 and Capan-2 as well as for the PSC lines RLT-PSC, hPSC21-S/T and hPSC128-S/V. In the control condition (black) cells are in direct cell-cell contact, while in the transwell condition (white) effector and target cells are separated by a 0.4 porous transwell membrane. Cytotoxicity was measured by flow cytometry after 4h coculture. Data is depicted as mean (± SEM) for five independent donors. *, p < 0.05. Wilcoxon Signed Ranks Test (2-tailed).

A transwell assay was performed to investigate the need for direct cell-to-cell contact between the (un)stimulated NK cells and the PCC or PSC in order to get killing. A statistically significant difference was observed for all cell lines between the transwell setting and the control setting where effector and target cells were in direct contact (Figure 2). Our data show that unstimulated as well as stimulated NK cells need direct cell-to-cell contact with the target cells, indicating that membrane factors play a major role to effectuate a cytotoxic effect of NK cells towards PCC and PSC cells. No killing was observed when effector and target cells were separated by a transwell.

### IL-15 results in upregulation of NKG2D and TIM-3 on NK cells from healthy donors

**Figure 3:**
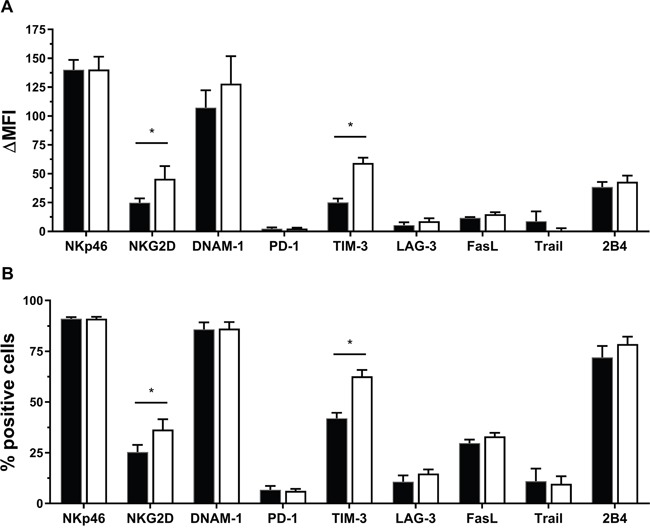
Effect of IL-15 on expression of different NK cell receptors **(A)** Differences in mean fluorescence intensity (ΔMFI) between the receptor and corresponding isotype are shown for both unstimulated (not stim.) and IL-15 stimulated NK cells. **(B)** Differences in percentage positive cells (% Overton) are shown for both unstimulated (not stim.) and IL-15 stimulated NK cells Analysis was performed with flow cytometry. Data is depicted as mean (± SEM) for eight donors (except for TRAIL, n = 7). *, p < 0.05. Wilcoxon Signed Ranks Test (2-tailed).

To unravel the mechanism via which IL-15 potentiates NK cells to kill PCC and PSC, the level of expression of a range of regulating molecules on both unstimulated and IL-15 stimulated NK cells was quantified. The receptors NKp46, NKG2D, DNAM-1, TIM-3 and 2B4 were all clearly expressed on unstimulated NK cells with ΔMFI levels above 25. PD-1, LAG-3, FasL and TRAIL were less expressed (ΔMFI levels ranging from 2 to 12). Only NKG2D (1.8-fold increase) and TIM-3 (2.4-fold increase) were statistically significantly increased after IL-15 stimulation (Figure 3A). Looking at the percentage of cells expressing the receptors, 70 to 91% of the unstimulated NK cells were positive for NKp46, DNAM-1 and 2B4; 25 to 41% were positive for NKG2D, TIM-3 and FasL; while PD-1, LAG-3 and TRAIL presence ranged from 6% to 25%. Similar to the MFI data, IL-15 induced a significant increase of NKG2D (1.4-fold) and TIM-3 (1.5-fold) positive NK cells (Figure 3B).

### NKG2D is partially responsible for the augmented killing of PCC and PSC by IL-15 stimulated NK cells

**Figure 4:**
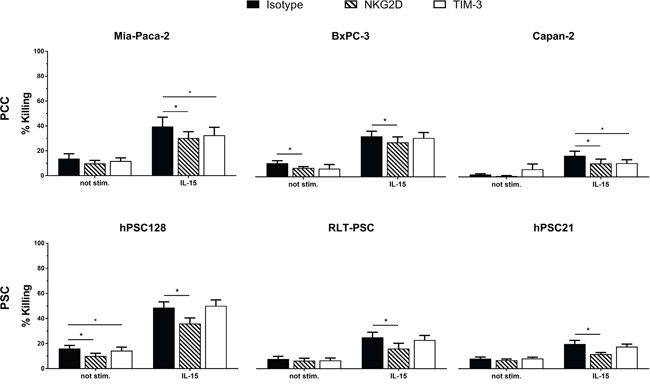
NKG2D and TIM-3 are partially responsible for IL-15 stimulated cytotoxic effect on PCC and PSC Percentages of killing are shown for PCC lines Mia-Paca-2, BxPC-3 and Capan-2, as well as for the PSC lines RLT-PSC, hPSC21-S/T and hPSC128-SV. Effectors cells were blocked with neutralizing NKG2D antibody (striated bars), TIM-3 antibody (white bars) or corresponding isotype antibody (black bars). Results were measured using a 4h flow cytometric assay. Data is depicted as mean (± SEM) for five independent donors. *, p < 0.05. Wilcoxon Signed Ranks Test (2-tailed).

We blocked NKG2D and TIM-3 using monoclonal neutralizing antibodies to investigate whether these proteins play a role in the higher killing rates after IL-15 stimulation. Blocking of NKG2D resulted in a statistically significant decrease of killing by IL-15 stimulated NK cells in all PCC and PSC (Figure 4). Remarkably TIM-3 neutralization resulted in a significant decrease in cytotoxicity for 2 PCC lines, Mia-Paca-2 and Capan-2. Within our PSC line panel, no significant difference in cytotoxicity was observed after blocking TIM-3 on IL-15 stimulated NK cells. Our data indicate that NKG2D is partially responsible for the augmented killing of PSC and PCC by IL-15 stimulated NK cells, while for TIM-3 this is only the case for PCC.

### IL-15 stimulated NK cells are capable of killing primary PSC in an autologous setting

**Figure 5:**
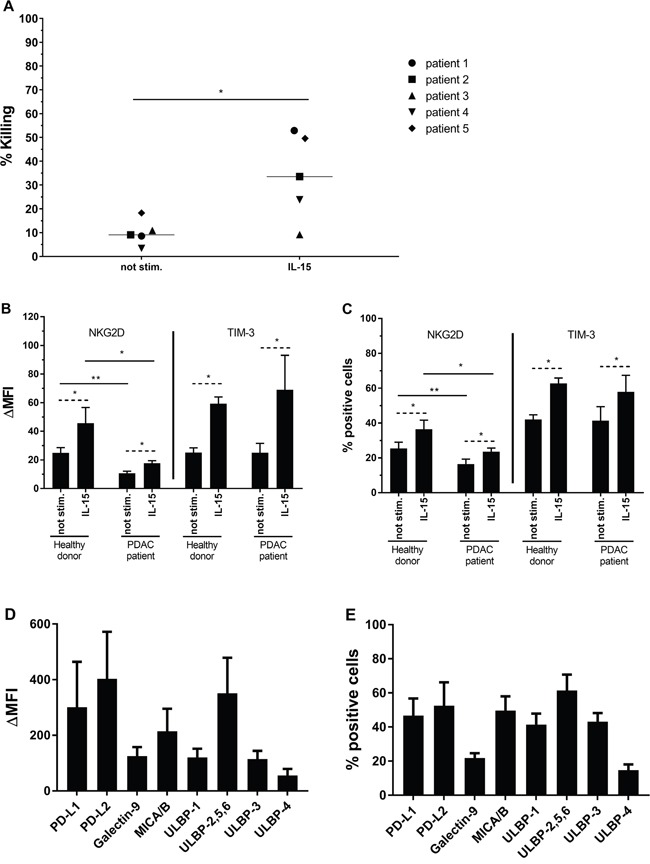
Results of PDAC patient experiments **(A)** Percentages of killing are shown for PSC from 5 PDAC patients after 4h coculture with (un)stimulated autologous NK cells. Results were measured using a flow cytometric analysis. Median is indicated. Wilcoxon Signed Ranks Test (1-tailed). **(B, C)** Expression **(B)** and percentage positive cells **(C)** for NKG2D and TIM-3 on (un)stimulated NK cells from healthy donors (n=8) or PDAC patients (n=4). Data is depicted as mean (± SEM). Wilcoxon Signed Ranks Test (1-tailed) is used within healthy donor or PDAC patients population (striated line). Mann-Whitney U test is used between healthy donor and PDAC patients population (full line). **(D, E)** Expression **(D)** and percentage positive cells **(E)** for ligands on primary PSC (n=6). Median is indicated. *, p < 0.05; **, p < 0.01.

As shown in Figure 5., we investigated the capacity of IL-15 stimulated NK cells to kill PSC in an autologous setting (Figure 5A). In 4 out of 5 PDAC patient samples tested, stimulation of NK cells with IL-15 resulted in a statistically significant increased capacity of killing of autologous PSC derived from tumor tissue, ranging from 23.8% to 52.9% killing after stimulation (2.7- to 6.9-fold increase; p < 0.05) at an E:T ratio of 5:1. Interestingly, these killing rates are similar compared with killing of PSC cell lines in an allogeneic setting (Figure 1, ranging from 18.3% to 50% for ratio 5:1). For patient 3, 10.9% of PSC were killed by unstimulated autologous NK cells with no further increase following IL-15 stimulation.

In analogy with healthy donor NK cells, the expression of NKG2D and TIM-3 on unstimulated versus IL-15 stimulated patient NK cells is significantly increased by 1.7- and 2.8-fold respectively (p<0.05 for both markers) (Figure 5B). Similarly, the percentage of PDAC patient NK cells positive for NKG2D and TIM-3 is significantly increased following IL-15 stimulation from 16.4% to 23.6% and from 41.2% to 57.9% positive NK cells, respectively (Figure 5C). NKG2D expression on patient NK cells was lower than on NK cells of healthy donors, also after IL-15 stimulation, whereas TIM-3 expression was comparable. A similar trend is seen for the percentage of NK cells expressing these two receptors (Figure 5B and 5C).

PSC derived from the PDAC tissue samples were evaluated for the NKG2D ligands MICA/B and different ULBPs, the TIM-3 ligand Galectin-9 and the PD-1 ligands PD-L1 and PD-L2. These ligands were all present on PSC, with ΔMFI ranging from 56 to 403 (Figure 5D) and the percentage of positive cells from 14 to 61% (Figure 5E). No correlation was found between the amount of expression of the ligands and the amount of killing of the PSC (data not shown).

## DISCUSSION

In this study we looked into the potential of (IL-15 stimulated) NK cells to kill PSC. Depletion of PSC and thus the stromal compartment has proven successful since the addition of the chemotherapeutic agent nab-Paclitaxel, the albumin-bound form of paclitaxel which accumulates in the tumor cells and stroma [28], results in better survival than chemotherapy alone [9, 29]. Although there is some evidence that depletion of the stroma might also cause faster tumor progression [30, 31], several studies showed that stromal depletion combined with immunomodulation had better outcomes than immunomodulation alone [30, 32]. Currently, several other strategies are being investigated in which stromal depletion is pursued, however none have explored the potential of NK cells [11, 33, 34]. To our knowledge, we are the first to describe NK cell-mediated killing of PSC.

In addition, we show that NK cells are capable of killing the PCC. Our results are in concordance with those of others who showed that NK cells from PDAC patients are capable of killing PCC lines, other than the ones used in this study [27, 35]. Although IL-15 has recently been acknowledged as a versatile molecule with great potential in cancer immunotherapy and NK cell stimulation [22, 36–38], none of these PCC studies evaluated IL-15 activated NK cells. IL-15 has already proven to have a beneficial effect in PDAC since Yoshida et al. showed that IL-15 producing PDAC cells induced a tumor reducing effect via NK cells [39] and Jing et al. showed that human umbilical cord blood-derived mesenchymal stem cells producing IL-15, eradicate pancreatic tumors in mice [40]. Furthermore, it had been demonstrated that NK-92, an IL-2 dependent human NK cell line, is able to kill several PCC lines. Here, a similar amount of killing for the CAPAN-2 cell line was observed but the Mia-Paca cell line was killed at least 40% less than with IL-15 stimulated NK cells evaluated in this study, stressing the potential of IL-15 [41]. IL-15 has proven safe for administration in humans in a first clinical trial [42]. These results mark an important milestone for IL-15 as a highly promising anticancer drug that has beneficial properties compared to IL-2. Treatment with high doses (due to the very short half-life) of IL-2 causes vascular leak syndrome, pulmonary edema, hypotension and heart toxicities, as well as expansion of the immunosuppressive Tregs [43]. IL-15 has a lower toxicity profile and does not stimulate expansion of Tregs. Moreover, a new stabilized IL-15 product, the superagonist ALT-803, has shown great potential in cancer [44, 45]. Currently several clinical trials are investigating IL-15 for cancer treatment of which one investigates ALT-803 in combination with gemcitabine and nab-Paclitaxel in PDAC (NCT02559674). In the phase IB/II trial, the maximum tolerated dose, safety profile and overall survival will be tested. Our data show that IL-15 has the potential to play an important role in the treatment of PDAC by stimulating NK cells to target both tumor cells and the surrounding desmoplastic barrier.

A hurdle to overcome for NK cell-mediated immunotherapy in PDAC is that NK cells have decreased activity in PDAC patients [46]. We observed a lower expression of the activating receptor NKG2D on NK cells from the PDAC patients as compared to healthy control NK cells. These results are in concordance with data presented by others [27, 47]. Our results also show that NKG2D is partially responsible for the mechanism via which PSC and PCC are killed by IL-15 stimulated NK cells. As demonstrated by Horng et al. in mice [48] in a non-PDAC context, IL-15 is capable of increasing NKG2D expression on patient NK cells. Furthermore, we observed an upregulation of TIM-3 on NK cells after IL-15 stimulation. This is in accordance with previous findings, also in a non-PDAC context [49]. Concerning the role of TIM-3 on NK cells, contradictory results have been published [50]. On the one hand, Ndhlovu et al. showed that cytotoxic capacity and IFN-γ production were associated with NK cells expressing the highest TIM-3 levels [49] and in accordance with our data, Gleason et al. demonstrated that TIM-3 was upregulated after NK cell activation and promoted IFN-γ production in response to its ligand Galectin-9 [51]. These results suggest that TIM-3 is a marker of fully functional and mature NK cells and has an activating role, in contrast with data of Ndhlovu et al., who demonstrated that NK cell-mediated cytotoxicity is suppressed when TIM-3 is activated by crosslinking antibodies or after encounter with target cells expressing Galectin-9 [49]. Accordingly, it has been shown that TIM-3 blockade improved NK cell-mediated cytotoxicity in human lung adenocarcinoma and advanced melanoma [52, 53]. We observed reduced killing of PCC after TIM-3 blockade which is in contradiction with the results of the latter. These data underscore the ambiguous role of TIM-3 on NK cell function in cancer and warrants further investigation.

As for the effect of IL-15 on the activating NK cell receptors NKp46, DNAM-1 and 2B4 (i.e. no changes), our observations are similar to the ones found by others [54, 55]. For the expression of FasL, we observed slightly higher expression than Feng et al., while they show strong upregulation of TRAIL which we did not observe [54]. These differences might be related to their longer IL-15 stimulation period of 10-12 days. Regarding LAG-3, we observed no upregulation of the protein. Although one study observed an upregulation in LAG-3 and PD-1 mRNA after IL-15 stimulation of NK cells, we are the first to describe the effect on LAG-3 on protein level [56].

In order to gain mechanistic insight in the susceptibility of PSC for NK cell-mediated killing, we screened PSC from the PDAC patients for NK cell ligands. Our novel observation of MICA/B expression on PSC of PDAC patients adds to the data of Duan et al., showing expression of MICA/B in serum and on tumor cells of PDAC patients [27]. Likewise, we show expression of ULBP 1,2,3,4,5 and 6 on primary PSC cells, adding to the data of Wrobel et al who demonstrated expression of ULBP 1,2,3 and 4 on two PCC lines [57]. Regarding the expression of Galectin-9, we are the first to describe the expression of this TIM-3 ligand on PSC. Moreover, we assessed the expression of PD-L1 and PD-L2, since it has already been shown that blockade of PD-L1 combined with inhibition of IL-6 produced by PSC has beneficial effects on PDAC in murine models [58]. We demonstrate both PD-L1 and PD-L2 surface expression on primary PSC using flow cytometry, which is in concordance with immunohistochemistry data shown by Nomi et al [59]. The expression of these ligands can be of interest as future points of action for novel immunotherapies that also aim to target the cancer cells as well as the shield made by the PSC.

## MATERIALS AND METHODS

### Ethics statement

This study was approved by the local Ethics Committee of the University of Antwerp (Antwerp, Belgium) under the reference number 14/47/480. Peripheral blood mononuclear cells (PBMC) were isolated from whole blood samples from patients with pancreatic ductal adenocarcinoma (PDAC) using a Ficoll density gradient centrifugation (Ficoll-Paque PLUS; GE Healthcare). From the same patients, tissue was obtained from the resected tumor after Whipple procedure. For healthy controls, PBMC were isolated from adult volunteer whole blood donations supplied by the Red Cross Flanders Blood Service (Mechelen, Belgium). Selection of the donors and collection of blood was performed according to Belgian law and Belgian Red Cross policy.

### Human (primary) cell lines

Three human pancreatic cancer cell (PCC) lines were used: *(1)* Mia-Paca-2 (DSMZ, Germany), cultured in Dulbecco's Modified Eagle Medium (DMEM) supplemented with 10% Fetal Bovine Serum (FBS), 2.5% Horse Serum and 2mM L-Glutamine (Thermo Fisher Scientific), *(2)* BxPC-3 (ATCC, USA) and *(3)* Capan-2 (ATCC, USA), both cultured in Roswell Park Memorial Institute (RPMI) 1640 supplemented with 10% FBS and 2mM L-Glutamine. Three human pancreatic stellate cell (PSC) lines were used: *(1)* RLT-PSC (established at the Faculty of Medicine of the University of Mannheim) [60], *(2)* hPSC21-S/T and *(3)* hPSC128-SV (both established at the Tohoku University Graduate School of Medicine) [61] are cultured in DMEM-F12 (1:1) supplemented with 10% FBS and 2mM L-Glutamine. Cell lines were split twice a week and incubated at 37°C with 5% CO_2_.

Primary PSC were cultured from human PDAC tissue samples using an outgrowth method [62]. Briefly, PDAC tissue samples were put in a sterile petri dish and cut in small pieces of 2-3 mm^3^ using a scalpel. Next, the tissue pieces were transferred to a 75 cm^2^ culture flask and incubated in DMEM-F12 supplemented with 10% FBS, 2mM L-Glutamine, 500 U/ml penicillin and 500 μg streptomycin. Culture medium was changed twice a week. After an average of 3 weeks, PSC spontaneously grew out of the tissue pieces. Cells were passaged using trypsin-EDTA and incubated at 37°C and 5% CO_2_. Characterization of the primary PSC was performed by checking expression of the following markers [63]: α-smooth muscle actin (α-SMA), glial fibrillary acidic protein (GFAP), Vimentin and Desmin using an immunohistochemistry (IHC) staining protocol as described before with minor modifications [60].

### NK cell isolation and stimulation

Cryopreserved PBMC where thawed and incubated overnight at 37°C and 5% CO_2_ in complete medium (RPMI 1640 supplemented with 10% FBS, 2mM L-Glutamine, 100 U/ml penicillin, 100 μg streptomycin and sodium-pyruvate). Subsequently, NK cells were isolated using negative magnetic activated cell sorting (MACS), according to the manufacturer's protocol (Miltenyi Biotec). After isolation, purity of the NK cells - measured by flow cytometric immunophenotyping the cells with CD3-FITC (Immunotools) and CD56-PE (BD Biosciences) – was above 90%. NK cells were split in 2 equal portions; one to stimulate with 10 ng/mL recombinant human IL-15, while the other portion was left untreated. Both conditions were incubated overnight at 37°C and 5% CO_2_.

### NK cell-mediated cytotoxicity assays

In order to measure the cytotoxic capacity of (un)stimulated peripheral blood NK cells towards PCC and PSC, a flow cytometric assay was used as described before with minor adjustments [64–66]. Briefly, PCC and PSC were labelled with the green fluorescent membrane dye PKH-67 (Sigma Aldrich) according to manufacturer's protocol and served as ‘target cells’. PKH-67-positive target cells were put in coculture with (un)stimulated effector NK cells at three different effector:target (E:T) ratio’s: 10:1, 5:1 and 1:1. In the *ex vivo* autologous experiments, only the 5:1 ratio was used. Tumor cells incubated without NK cells served as controls.

The necessity of direct cell-cell contact between target and effector cells was investigated by using a transwell assay which prevented direct contact. PKH-67 labelled target cells were put in the bottom compartment of a 96-well transwell plate (HTS Transwell®-96 Well, Pore size 0.4μm, Corning) and (un)stimulated target cells were added in the top compartment at an E:T ratio of 5:1. Cocultures of effector and target cells with direct cell-cell contact served as positive controls while cultures of tumor cells without effector cells served as negative controls.

In a specific set of experiments, the involvement of NKG2D and TIM-3 in NK cell-mediated killing of PCC and PSC was measured by a 2h preincubation of (un)stimulated NK cells with 20 mg/ml anti-NKG2D (R&D Systems), anti-TIM-3 (eBioscience) or corresponding isotype control antibodies (Jackson Laboratories) prior to coculture at an E:T ratio of 5:1.

In all experiments, cocultures incubated 4h after which the supernatant was discarded and cells were acquired on the FACS Aria II (BD Biosciences) after Annexin V-APC (BD Biosciences) and propidium iodide (PI) (Thermo Fisher Scientific) staining for quantification of the target cell viability. Target cell killing by NK cells was calculated by the following formula: % killing=100%−(% Annexin V−/PI− tumor cells with NK cells% Annexin V−/PI− tumour cells without NK cells)×100 [64].

### Phenotyping of target and effector cells

The immunophenotype of purified (un)stimulated NK cells was evaluated using PE-conjugated antibodies (BD Biosciences) and flow cytometry for the following surface markers that might have a stimulating or inhibiting effect on NK cell functions: NKG2D, DNAM-1, PD-1, TIM-3, LAG-3, NKp46, FasL, TRAIL and 2B4. In addition, corresponding ligands on the PCC and (primary) PSC were measured using PE-conjugated antibodies and flow cytometry for the following markers: MICA/B, ULBP1, ULBP2,5,6, ULBP3, ULBP4 (NKG2D ligands; antibodies all from R&D Systems), Galectin-9 (TIM-3 ligand; antibody from BD Biosciences), PD-L1 and PD-L2 (PD-1 ligands; antibodies both from BD Biosciences). Corresponding isotype controls were included for all samples and served as negative controls. All samples were measured on a FACSCan flow cytometer (BD Biosciences).

### Data analysis

All flow cytometry data were analyzed using FlowJo v10.1. GraphPad Prism 7 was used for data comparison and artwork. Statistical analyses were performed with IBM SPSS Statistics 23. Paired-wise non-parametric Wilcoxon signed ranks testing was performed to compare means. Correlations were investigated using the Spearman's correlation coefficient. Differences were predefined to be considered as statistically significant when p < 0.05.

## CONCLUSION

We show that NK cells are capable of killing both PCC and PSC and this significantly better after stimulation with IL-15. These results support the importance and value of an IL-15 and NK cell orientated approach to treat PDAC. NKG2D is partially responsible for this effect, as well as TIM-3 to a lesser degree. Our observations regarding the expression and presence of several NK cell ligands on primary PSC reveal possible future targets to tackle remaining PSC. Further research to investigate the *in vivo* potential of this new immunotherapeutic approach in PDAC will be performed in the near future.
